# Election of Officers

**Published:** 1874-04

**Authors:** 


### Election of Officers.

At the Annual Meeting of the Buffalo Medical Association, April, the following officers were elected.

*President.*—JAMES P. WHITE, M. D.

*Vice President.*—WILLIAM. GOULD, M. D.

*Secretary.*—EDWIN R. BARNES, M. D.

*Treasurer.*—J. J WALSH, M· D

*Librarian.*—P. H. STRONG' M. D.

The meeting was largely attended and was full of interest. We hope in our next issue to give an abstract of the proceedings.

